# Validation of a point-of-care instrument for bedside glutamine screening in the intensive care unit

**DOI:** 10.1186/2197-425X-3-S1-A190

**Published:** 2015-10-01

**Authors:** L Pettersson, S Ryden, I Tjäder, O Rooyackers, J Wernerman

**Affiliations:** Karolinska University Hospital, Huddinge, Sweden; Karolinska Institutet, CLINTEC, Huddinge, Sweden

## Introduction

Glutamine supplementation for critically ill patients has become controversial after trials showing a negative effect on clinical outcome. However, in addition to patients with very low glutamine levels there are patients with very high levels and most likely supplementation needs to be individualised to the patients with low levels. Unfortunately no clinically acceptable bedside analysis for glutamine is available.

## Objectives

In this study, a point-of-care instrument developed for measuring glutamine levels in cell cultures was validated for bedside use in the ICU setting and compared with a standard HPLC technique to measure plasma glutamine. The aim was to make evaluations for absolute measurements and for clinical screening purposes.

## Methods

Blood samples were obtained during ICU stay from one hundred adult ICU patients 3-5 days apart as long as patients were treated in the ICU. Patients younger than 18 years, not given consent or re-admitted to the ICU were excluded. Each sample was divided into 3 aliquots; for analyses of plasma and whole blood glutamine by the point-of-care (POC) instrument (Bioprofile Basic, Nova Biomedical, Waltham, MA, USA) and for analysis of plasma glutamine concentration by the gold standard HPLC technique. The analyses were compared with regression analyses and Bland-Altman plots.

## Results

The initial sample of all subject (n = 100) showed a good correlation between plasma and whole blood analyses on the POC when corrected for hematocrit (r = 0.99, p < 0.001). Comparison of plasma glutamine measured on the POC and HPLC gave a good correlation (r = 0.98, p < 0.001) but Bland-Altman analyses revealed a line of identity of -222 µmol /L and lines of agreement (2xSD) of 55 and -499 µmol /L. However, after pragmatic adjustments for the constant bias (222 µmol /L) and hematocrit (0.30), POC analyses of whole blood compared with HPLC plasma analyses (all samples; n = 316) showed a line of identity of -34 µmol /L and limits of agreement between 288 and -355 µmol /L.

## Conclusions

When compared to the HPLC gold standard, in particular the bias of -222 µmol /L, indicates that the point-of-care instrument is not suitable for absolute plasma glutamine concentration measurements. For screening purposes to identify patients with hypoglutaminemia and hyperglutaminemia the instrument may be useful when adjusted for this constant bias. Identification of subjects with hypoglutaminemia for supplementation was safe. The point-of-care instrument may also serve to screen for scientific studies.

## Grant Acknowledgment

Stockholm County Council.

